# Study on the Effect of 4D-CT Special Reconstruction Images for Evaluation of the Cardiac Structure Dose in Radiotherapy for Breast Cancer

**DOI:** 10.3389/fonc.2020.00433

**Published:** 2020-03-31

**Authors:** Ming Su, Guanzhong Gong, Xiaoping Qiu, Ying Tong, Qian Li, Yong Yin

**Affiliations:** ^1^School of Nuclear Science and Technology, University of South China, Hengyang, China; ^2^Shandong Cancer Hospital and Institute, Shandong First Medical University and Shandong Academy of Medical Sciences, Jinan, China

**Keywords:** 4D-CT, dose accumulation, special reconstruction, breast cancer, radiotherapy, cardiac structure

## Abstract

**Objective:** To study the dosimetric effect on special reconstruction images obtained from an electrocardiograph-gated four-dimensional computed tomography (ECG 4D-CT) series and compare it with the accumulation dose assessment of ECG 4D-CT.

**Methods:** Fifteen patients underwent ECG 4D-CT scans to obtain a 4D-CT series. The 20 phase images of 0–95% were reconstructed at intervals of 5% of the cardiac cycle by the 4D-CT series. The 4D-CT series was specially reconstructed, and the maximum intensity projection (MIP), minimum intensity projection (MinIP), average intensity projection (AIP), and sum intensity projection (SIP) were obtained. The left ventricular muscle (LV) and the anterior descending branch of the left coronary artery (LAD) were delineated on all series. The intensity-modulated radiation therapy (IMRT) plan for left breast cancer was designed on the basis of the 0% phase, and the accumulative dose (Dose_−acc_) of 20 phases was obtained by deformation registration. The dose-volume indexes of the LV and LAD were compared based on different CT series.

**Results:** The dose-volume indices of V_5_, V_30_, V_40_, D_max_, and D_mean_ of the LV on MIP images were 3.8, 2.0, 0.9, 3.8, and 1.7%, respectively (relative to the Dose_−acc_). There was no significant difference in V_5_ or D_max_ between the MIP and Dose_−acc_ (*P* > 0.05). The change rates of D_max_ on the MinIP, SIP, and AIP images were 2.5, 3.1, and 1.5%, respectively (relative to the Dose_−acc_) (*P* < 0.05).

**Conclusion:** In the dose-volume evaluation of the LV, V_30_, V_40_, and D_mean_ obtained by MIP were essentially the same as those obtained by the Dose_−acc_ and can be used instead of the 4D-CT series to evaluate dose-volume indexes.

## Introduction

Breast cancer accounts for 24.2% of new cancer cases and 15.0% of cancer-related deaths in females. Globally, the incidence and mortality of breast cancer rank first in 154 and 104 countries, respectively (1). Radiation therapy (RT) is considered a curative-intent treatment for patients with breast cancer but can cause late locoregional complications such as cardiac toxicity, especially for left breast cancer.

Radiation-induced heart damage in breast cancer has received much attention. In a recent analysis, Bedi et al. (2) aimed to study the differences between treatment plans based on conventional and time-resolved four-dimensional CT. The results showed that heart movement had a greater impact on breast cancer radiotherapy than respiratory movement. The radiation treatment plan based on three-dimensional CT scan well-reflected the dose distribution in the four-dimensional CT derived data set. Using a three-dimensional computerized tomography (3D-CT) plan, Budrukkar et al. (3) prospectively investigated whether the 3D-CT plan was excellent in terms of long-term outcomes. However, static 3D-CT does not consider the effects of breathing and heartbeat movements on the location and shape of the heart, and heartbeat movement is the main factor affecting the inaccurate dose evaluation of the heart and its substructure in left breast cancer radiotherapy (4, 5). Electrocardiograph-gated four-dimensional computed tomography (ECG 4D-CT) provides better image quality and can reduce artifacts caused by the heartbeat motion. Moreover, contrast enhancement in ECG 4D-CT is helpful for identifying the extent of tissues, which can reduce errors in organ delineation (6). ECG 4D-CT is therefore considered to be a reliable and effective tool for assessing tumor and organ motion (7).

The prediction of radiation-induced cardiac injury is assessed by mainly dose-volume indices. ECG 4D-CT provides a basis for the accurate assessment of cardiac acceptance. However, the number of ECG 4D-CT images is large, so special reconstruction can be carried out to reduce the number of images. Whether special reconstruction images can accurately assess cardiac volume requires further exploration. In this study, we explored the accuracy of ECG 4D-CT special reconstruction images of left breast cancer in assessing cardiac acceptance. In addition, we compared the cumulative dose of ECG 4D-CT with that of special reconstruction images in dose assessment.

## Materials and Methods

### Materials

Fifteen female patients (aged 32–65 years, with a median age of 49 years) admitted to Shandong Cancer Hospital between June 14, 2016 and November 15, 2017, were selected as subjects. All patients had no contraindications to radiotherapy, and all patients were treated with intensity-modulated radiation therapy (IMRT).

### ECG 4D-CT Scanning and Reconstruction Image Acquisition

Under helical mode, all patients underwent enhanced ECG 4D-CT scans at the end of deep inspiratory breath holding (DIBH) with a Siemens dual source CT scanner (Siemens, SOMATOM Defintion Flash, Germany). At the end of the scan, 20 phases of 0–95% were reconstructed at an interval of 5% of the heart cycle. The CT image matrix size was 512 × 512, the thickness was 0.75 mm, and the interval was 0.5 mm. The ECG 4D-CT images were transferred to MIM Maestro (Version 6.6.9, MIM, USA) for maximum intensity projection (MIP), minimum intensity projection (MinIP), average intensity projection (AIP), and sum intensity projection (SIP) image reconstruction.

### Radiotherapy Design

The 4D-CT images were imported into the Eclipse 13.6 planning system (Varian Medical System, USA), and the IMRT plan was designed for chest wall, supraclavicular lymph node drainage area and armpit area of each patients with stage of T3–T4, based on the 0% phase image of each patient. All patients were treated with a 7-field IMRT plan, 2 Gy/Fx, for a total of 50 Gy/25Fx. The typical gantry angle configurations were 0°, 30°, 110°, 125°, 150°, 310°, and 330°.

### Delineating the Structure of the Heart

**Figure 1:**
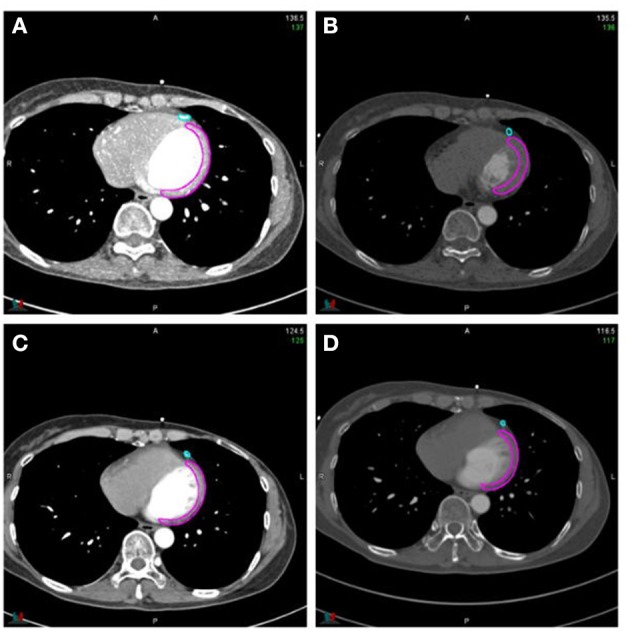
Reconstruction sketch. **(A)** MIP; **(B)** MinIP; **(C)** AIP; **(D)** SIP.

The 4D-CT series was transmitted to MIM software for 20 phases and special reconstruction image delineation and fusion. The left coronary artery (LAD) and left ventricular muscle (LV) were contoured in all 20 phases. The range of the LV was from the parietal plane of the left ventricle to the apex of the heart, excluding the interventricular septum, and the range of the LAD was from the bifurcation of the left main coronary artery to the apex of the heart (see Figure 1 for details). When delineating window width (WW)/window level (WL) was 400/40 HU, and WW/WL was slightly adjusted when delineating on special reconstructed images. All the cardiac region of interest (ROI) delineations were completed by 3 radiation oncologists reviewing and browsing the series of each patient and then jointly developing a delineation standard.

### Deformation Registration

**Figure 2:**
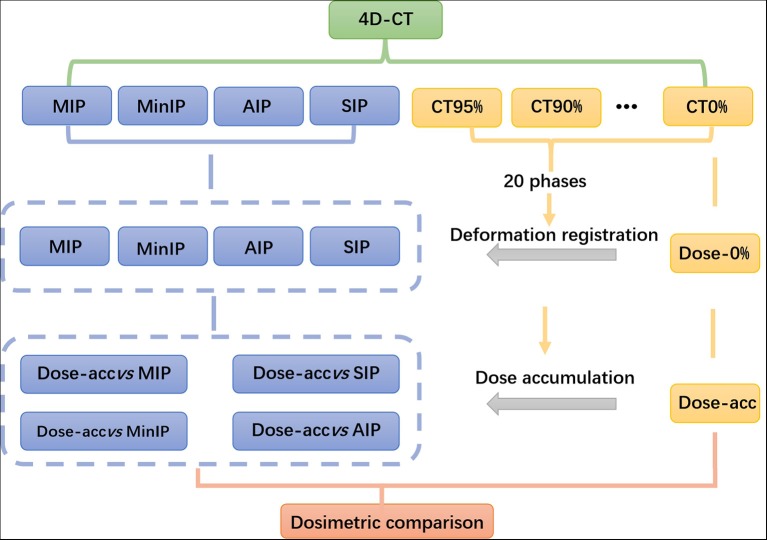
Dosimetric comparison flow chart.

The radiotherapy plan was introduced into MIM software, and the dose of the 0% phase was mapped to the remaining 19 phases of the 4D-CT series. The dose of the remaining 19 phases was deformed to that of the 0% phase, and the cumulative dose of the 4D-CT series was obtained, which was named the accumulative dose (Dose-acc). The dose deformation of the 0% phase was registered into MIP, MinIP, SIP and AIP images. The flow chart is shown in Figure 2.

### Data Statistics

The dose-volume indexes of the LV and LAD, including V_5_, V_10_, V_20_, V_30_, V_40_, D_min_, D_max_, and D_mean_, were calculated on the MIP, MinIP, SIP, and AIP images and 4D-CT series, and statistical analyses on the volume of the LV and LAD were performed on all series. Analyses were carried out using SPSS 17.0 software, and *P* < 0.05 was considered significant.

## Results

### Volume Changes in the LV and LAD

➀ The volume of the LV showed a decreasing trend on 4D-CT, MinIP, AIP, MIP, and SIP images: (54.55 ± 12.68) cm^3^, (53.36 ± 12.08) cm^3^, (52.05 ± 13.31) cm^3^, (50.92 ± 11.10) cm^3^, and (42.37 ± 9.72) cm^3^, respectively. There was a significant difference between the SIP images and the 4D-CT series (*P* < 0.05).

➁ The volume of the LAD showed a decreasing trend on MIP, AIP, SIP, MinIP, and 4D-CT images: (3.40 ± 0.94) cm^3^, (2.24 ± 0.81) cm^3^, (1.97 ± 0.86) cm^3^, (1.67 ± 0.47) cm^3^, and (1.45 ± 0.24) cm^3^, respectively (*P* < 0.05). The details are shown in Table 1.

**Table 1:** Volume changes in the LV and LAD ($\bar{x}$ ± s) cm^3^.

**Organ**	**4D-CT**	**MinIP**	**AIP**	**MIP**	**SIP**	***P*_**1**_**	***P*_**2**_**	***P*_**3**_**	***P*_**4**_**
LV	54.55 ± 12.68	53.36 ± 12.08	52.05 ± 13.31	50.92 ± 11.10	42.37 ± 9.72	0.217	0.157	0.601	0.004
LAD	1.45 ± 0.24	1.67 ± 0.47	2.24 ± 0.81	3.40 ± 0.94	1.97 ± 0.86	0.001	0.001	0.047	0.011

*P_1_–P_4_ represent MIP, AIP, MinIP, and SIP images, respectively, values were derived from the Wilcoxon non-parametric test*.

### Dosimetric Changes in the LV

In the dosimetric comparison of the LV, the results showed that regardless of the kind of special reconstruction method, half of the dose-volume indexes were not statistically significant compared with the Dose_−acc_ (*P* > 0.05). In the MIP images, the change rates of V_10_, V_20_, V_30_, V_40_, D_min_, and D_mean_ were 6.8, 5.1, 2.0, 0.9, 10.5, and 1.7%, respectively, and the smallest change rate was that of V_40_ (0.9%). In the AIP images, the change rates of V_20_, V_30_, and D_mean_ were 17.6, 13.5, and 6.2%, respectively, and the smallest change rate was that of D_mean_ (6.2%). However, in MinIP and SIP images, the change rates were larger than those in the MIP and AIP images (2.5–19.9% on MinIP images and 3.1–14.0% on SIP images), and only V_10_ and V_20_ were not statistically significant compared with the Dose_−acc_ (*P* > 0.05). The details are shown in Table 2.

**Table 2:** Image changes in the dose-volume indexes of the LV ($\bar{x}$ ± s) %.

**LV**	**V_**5**_**	**V_**10**_**	**V_**20**_**	**V_**30**_**	**V_**40**_**	**D_**max**_**	**D_**min**_**	**D_**mean**_**
MIP	57.26 ± 22.87	38.47 ± 24.36	27.07 ± 19.14	22.69 ± 18.17	13.51 ± 11.86	48.44 ± 4.14	1.72 ± 2.2	14.55 ± 7.06
AIP	59.33 ± 22.62	39.22 ± 24.15	27.99 ± 18.65	23.32 ± 17.79	14.76 ± 10.93	47.52 ± 5.09	1.68 ± 2.02	14.88 ± 6.75
MinIP	59.80 ± 25.80	41.66 ± 23.21	30.27 ± 18.34	24.93 ± 16.81	17.11 ± 12.82	48.36 ± 4.04	1.75 ± 1.85	14.40 ± 7.16
SIP	62.45 ± 22.29	41.67 ± 24.39	31.59 ± 20.60	25.65 ± 17.86	16.58 ± 12.41	49.11 ± 3.92	1.69 ± 2.04	15.12 ± 5.83
Dose_−acc_	51.10 ± 24.88	31.16 ± 18.45	22.55 ± 16.50	18.18 ± 16.47	13.65 ± 15.56	46.66 ± 5.64	1.55 ± 1.94	12.87 ± 7.70
*P_1_*	0.003	0.191	0.211	0.156	0.363	0.011	0.116	0.053
*P_2_*	0.022	0.041	0.191	0.078	0.024	0.020	0.036	0.125
*P_3_*	0.005	0.053	0.112	0.031	0.035	0.009	0.002	0.394
*P_4_*	0.001	0.088	0.061	0.036	0.047	0.011	0.044	0.027

P_*1*_–P_*4*_ represent the Dose_−*acc*_ and MIP, AIP, MinIP, and SIP images; values were derived from the Wilcoxon non-parametric test.

### Dosimetric Changes in the LAD

Contrary to the LV, the dose-volume indices of the LAD were significantly different from those of the Dose_−acc_ in four special reconstruction images. In the MIP images, the change rates of V_5_, V_10_, V_20_, V_30_, V_40_, D_max_, D_min_, and D_mean_ were 3.6–19.2%, and the smallest change rate was that of D_max_; in the AIP images, the change rates of the above indexes were 3.0–28.1%, and the smallest change rate was that of D_max_; in the MinIP images, the change rates were 2.0–18.5%, and the smallest change rate was that of D_max_; in the SIP images, the change rates were 3.4–20.4%, and the smallest change rate was that of D_max_. The details are shown in Table 3.

**Table 3:** Dose-volume changes in the left anterior descending coronary artery ($\bar{x}$ ± s) %.

**LAD**	**V_**5**_**	**V_**10**_**	**V_**20**_**	**V_**30**_**	**V_**40**_**	**D_**max**_**	**D_**min**_**	**D_**mean**_**
MIP	79.15 ± 18.45	65.88 ± 17.13	57.48 ± 16.32	48.01 ± 17.05	39.13 ± 12.52	48.04 ± 6.01	2.90 ± 2.31	25.39 ± 9.07
AIP	80.60 ± 16.20	67.96 ± 15.53	59.16 ± 14.38	48.91 ± 13.49	39.68 ± 10.90	48.04 ± 7.10	2.84 ± 2.31	25.17 ± 9.65
MinIP	82.09 ± 15.22	69.87 ± 12.27	60.21 ± 14.97	49.19 ± 16.85	39.91 ± 20.48	47.00 ± 5.79	3.84 ± 3.95	27.32 ± 11.0
SIP	82.09 ± 13.35	66.78 ± 14.42	57.49 ± 13.43	46.87 ± 14.37	38.65 ± 14.06	48.98 ± 4.15	2.94 ± 2.30	25.99 ± 8.68
Dose_−acc_	72.65 ± 21.56	54.97 ± 20.44	44.05 ± 19.15	37.14 ± 20.41	29.26 ± 22.01	46.07 ± 7.72	2.62 ± 2.22	21.82 ± 8.31
*P*_1_	0.004	0.002	0.011	0.017	0.002	0.015	0.016	0.011
*P*_2_	0.001	0.001	0.020	0.006	0.001	0.001	0.013	0.003
*P*_3_	0.006	0.002	0.020	0.047	0.036	0.041	0.001	0.031
*P*_4_	0.001	0.008	0.012	0.036	0.009	0.004	0.002	0.027

*P_1_–P_4_ represent the Dose_−acc_ and MIP, AIP, MinIP, and SIP images; values were derived from the Wilcoxon non-parametric test*.

## Discussion

Heart injury is one of the main complications of radiotherapy for left breast cancer (8, 9). Mcgale et al. (10) showed that radiotherapy for left breast cancer increased the probability of ischemic heart disease, pericarditis and valvular disease. Darby et al. (11) showed that in radiotherapy for breast cancer, the average cardiac dose was positively correlated with the risk of coronary artery disease, and the risk of coronary artery injury increased by 7.4% with the increase of cardiac dose by 1 Gy. The study of Darby et al. (11) also showed that radiotherapy for left breast cancer increased the risk of ischemic heart disease, pericarditis, and valvular disease. Therefore, in radiotherapy for left breast cancer, an accurate assessment of the cardiac dose and its substructure is helpful for accurately predicting the occurrence probability of radiation-induced cardiac injury and reduce the injury by adjusting the radiotherapy plan or clinical intervention. It is also helpful for improving the quality of life and survival rate of patients.

In radiotherapy for breast cancer, especially left breast cancer, heartbeat movement is the main cause of an inaccurate dose assessment of the heart and its substructures (12, 13). A number of scholars have shown that deep inspiratory breath holding (DIBH) and autonomous breath control (ABC) techniques can reduce exposure from breast cancer radiotherapy (14–17).

However, Kataria et al. (5) proposed that in addition to respiratory movement, cardiac activity is also an important factor affecting the accuracy of treatment. The ECG 4D-CT series can accurately reflect the movement information of the tumor target area and organs endangered by dynamically tracking the movement of the heart and its substructures, providing a basis for precise radiotherapy. However, the 4D-CT series is generally divided into 20 phases, with 180–250 images per phase and 3,600–5,000 images per patient. Therefore, it is difficult to draw target areas and organs at risk (OARs) based on this series, and the drawing error will increase, which will affect the accuracy of the cardiac dose-volume index assessment to a certain extent. Special reconstruction projects the MIP, MinIP, AIP, and SIP images of the 4D-CT series and after special reconstruction, the number of series is greatly reduced, and the time and workload needed to draw target areas and OARs by doctors are reduced to 1/20 of the original. The MIP image generates a projection line according to the target direction observed by the operator. The maximum intensity voxel value of the projection line is taken as the signal intensity value of the generated image. The goal of projection image reconstruction is to remove the low-density areas. The MinIP, AIP, and SIP are, in turn, the minimum, average and sum of the intensity voxel values of the projection line as the signal intensity value of the resulting image. Because these four special reconstruction algorithms have their own characteristics, there are differences in the sizes and shapes of the target areas and the OARs delineated from the reconstruction images.

During the ECG 4D-CT scan, all patients were injected with contrast medium for enhancement in this study. With the use of contrast agents, the LV is relatively simple to delineate, and the reconstruction accuracy of its special reconstruction image is high, which can essentially reflect movement and morphological changes in the LV. In this study, it was also confirmed that the volume of the LV on 4D-CT was less than that on the MinIP, AIP, and MIP images. The volume of the LV on the 4D-CT series and on the MinIP, AIP, and MIP images was (54.55 ± 12.68) cm^3^, and (53.36 ± 12.08) cm^3^, (52.05 ± 13.31) cm^3^, and (50.92 ± 9.72) cm^3^, respectively. The above data show that when the LV was delineated by the MinIP, AIP, and MIP images, the contrast agent had a negligible effect on the volume and shape of the LV. However, the SIP image uses all the intensity voxels of the projection line as the signal intensity value of the resulting image. Because of the influence of contrast medium, the density of the cardiac vessels was relatively high, and the density of the LV was relatively low, so a portion of the LV was submerged by the blood-rich cardiac vessels; therefore, the volume of the LV on the SIP image differs greatly from that on the 4D-CT series (only 42.37 ± 9.72 cm^3^), so the SIP image cannot replace the 4D-CT series. In the evaluation of the dose-volume index of the LV, it was also confirmed that there were some differences in the evaluation of the dose-volume index because of the change in its volume and morphology. Among the four special reconstruction images, the dose-volume index of the MIP image differed slightly from that of the Dose_−acc_, reflected mainly in the change rate of V_5_, V_30_, V_40_, and D_mean_ being <5%. Except for the difference in V_5_ between the two groups, the other indexes had no statistical significance (*P* > 0.05). On MinIP and SIP images, only the change rates of D_max_ and D_mean_ were <5% compared with the Dose_−acc_; on the AIP images, only the change rates of D_max_ were <5% compared with Dose_−acc_. Although the LV volume on the MinIP and AIP images was not significantly different from that on the 4D-CT series, there was a large difference in the evaluation of the dose-volume index; therefore, it is not suitable to replace the 4D-CT series with AIP images or MinIP images. For the MIP images, the volume of the LV was close to that on the 4D-CT series, and the dose-volume indexes V_30_, V_40_, and D_mean_ of the LV were slightly different from those of the Dose_−acc_. Therefore, MIP images can be used instead of Dose_−acc_ to evaluate the dose-volume index of the LV.

When doctors delineated the LAD, they mainly outlined the contrast medium in the lumen of the LAD. However, the density of the contrast medium was higher than that of other cardiac tissues, which had a great influence on the special reconstruction of the LAD. This study also showed that in the evaluation of the dose-volume index of the LAD in the four special reconstruction algorithms, only the change rate of D_max_ in the LAD was <5% compared with the Dose_−acc_, but the difference was statistically significant (*P* < 0.05). Therefore, it is necessary to be careful when using the special reconstruction algorithm to evaluate the dose-volume index of the LAD. Although the difference between D_max_ and the Dose_−acc_ in the four special reconstruction algorithms was small, it was statistically significant (*P* > 0.05), so we do not recommend its use.

## Conclusion

In this study, MIP, AIP, MinIP, and SIP images were reconstructed, and the LV and LAD were outlined on these images and compared with the dose parameters of 4D-CT after accumulation. The results showed that the dose-volume parameters of the LV evaluated on MIP images had the smallest difference compared with those evaluated on 4D-CT. Therefore, MIP images can be used to evaluate the dose-volume parameter of the LV in radiotherapy. However, for the LAD, the difference was large, reaching 28.1%. Therefore, special reconstruction images cannot be used to replace 4D-CT images for LAD dosimetry evaluation.

## Data Availability Statement

The datasets analyzed in this article are not publicly available. Requests to access the datasets should be directed to suming1115@hotmail.com.

## Ethics Statement

This retrospective study was approved by the Institutional Ethics Review Board of the Shandong Cancer Hospital (No. 201809021). Medical record review was performed in accordance with Institutional Ethics Review Board guidelines.

## Author Contributions

YY contributed to the conception of the study. GG contributed significantly to analysis and manuscript preparation. MS performed the data analyses and wrote the manuscript. YT and QL helped perform the analysis with constructive discussions. XQ guides the writing of the article.

### Conflict of Interest

The authors declare that the research was conducted in the absence of any commercial or financial relationships that could be construed as a potential conflict of interest.
